# Role of Renin–Angiotensin System Inhibitors After Transcatheter Aortic Valve Replacement: A Systematic Review and Meta-analysis

**DOI:** 10.1097/MJT.0000000000001897

**Published:** 2025-01-29

**Authors:** Muhammad Burhan, Sahaab Noor, Mushood Ahmed, Saira Shafiq, Raheel Ahmed, Farhan Shahid

**Affiliations:** 1 Rawalpindi Medical University, Rawalpindi, Pakistan; 2 Department of Cardiology, Royal Brompton Hospital, London, United Kingdom; 3 National Heart and Lung Institute, Imperial College London, United Kingdom; 4 Department of Interventional Cardiology, Queen Elizabeth Hospital, Birmingham, United Kingdom

## Abstract

Supplemental Digital Content is Available in the Text.

***To the Editor:***

Aortic stenosis is a leading cardiovascular condition that can cause left ventricular hypertrophy because of sustained pressure overload.^1^ This structural change, seemingly associated with renin–angiotensin system (RAS) activation, can increase the risk of heart failure and contribute to diastolic dysfunction.^2^ Transcatheter aortic valve replacement (TAVR) is an effective intervention to treat severe aortic stenosis. In patients with severe ventricular remodeling after TAVR, RAS inhibitors (angiotensin-converting enzyme inhibitors and angiotensin receptor blockers) have been used as an effective treatment to mitigate the adverse effects.^3^ RAS inhibitors have demonstrated efficacy in limiting adverse ventricular remodeling, reducing left ventricular hypertrophy, and preventing myocardial fibrosis.^4^ Previous analyses on the role of RAS inhibitors after TAVR are mostly based on observational studies, limiting their generalizability. However, the evidence has become more robust with the addition of the recent RASTAVI trial.^5^ This has allowed a more comprehensive reassessment of the effectiveness of RAS inhibitors after TAVR.

The guidelines by the Preferred Reporting Items for Systematic Reviews and Meta-Analyses and Cochrane Handbook were used to report this systematic review and meta-analysis.^6,7^ A comprehensive and systematic search was formulated and conducted across PubMed, EMBASE, and the COCHRANE Library, with all relevant articles searched from database inception till October 2024 (see **Table, Supplemental Digital Content 1**, http://links.lww.com/AJT/A210). We included those studies that compared the use of RAS inhibitors after TAVR with those that did not use RAS inhibitors. We included randomized controlled trials and cohort studies and applied no restrictions on language, follow-up duration, or sample size. The primary outcome was all-cause mortality. The secondary outcomes were cardiovascular mortality, heart failure, major bleeding, aortic and mitral regurgitation, acute kidney injury, and stroke/transient ischemic attack (TIA). We used the Risk of Bias in Nonrandomized Studies of Interventions tool to appraise the quality of included nonrandomized studies.^8^ The Cochrane Risk of Bias tool (RoB 2.0) was used to assess the quality of randomized controlled trials.^9^ Statistical analysis was performed using R Studio's *meta* and *dmetar* packages. The dichotomous variables were pooled using odds ratio (OR) and its 95% confidence interval (95% CI) using the random-effects model. Heterogeneity was calculated using Higgins *I*^2^ statistic and visualized using a forest plot.^10^ Leave-one-out sensitivity analysis was performed to determine the effect of each study on *I*^2^ and effect size. Subgroup analysis and meta-regression were performed to explain heterogeneity across studies. Publication bias was visualized using a funnel plot, and Egger linear regression test was performed for quantitative assessment of outcomes reported by more than 10 studies. A *P* value of less than 0.05 was considered significant for all analyses.

A total of 13 studies^5,11–22^ were included in our systematic review, and only 1 study, which did not report the required outcomes, was not pooled in our meta-analysis.^11^ All the studies were cohort studies except one^5^ randomized controlled trial. A detailed study selection process is summarized in the Preferred Reporting Items for Systematic Reviews and Meta-Analyses flowchart (see **Figure, Supplemental Digital Content 1**, http://links.lww.com/AJT/A210). Thirty-six thousand five hundred thirteen patients were pooled in our analysis, from which 17,229 patients were in the RAS inhibitor group, and 19,284 patients were in the control group. Details regarding the baseline characteristics are provided in Table 1. Moderate risk of bias was observed in 5 studies^11,16,17,19,20^ primarily because of possible selective reporting of results (see **Figures 2, 3, Supplemental Digital Content 1**, http://links.lww.com/AJT/A210) The use of RAS inhibitors was significantly associated with a lower risk of all-cause mortality (OR 0.67; 95% CI, 0.59–0.77; *P* < 0.001; *I*^2^ = 67%) (Figure 1A) as compared to the control group, which was reported by 11 studies. Leave-one-out sensitivity analysis showed that no study significantly affected the pooled effect size and heterogeneity (see **Figure 4, Supplemental Digital Content 1**, http://links.lww.com/AJT/A210) The subgroup analysis was performed based on follow-up duration in years, which was insignificant (*P* = 0.18) (see **Figure 5, Supplemental Digital Content 1**, http://links.lww.com/AJT/A210). Univariate meta-regression analysis of included studies showed that publication year (*z* = −0.9, *P* = 0.3), age (*z* = −0.3, *P* = 0.74), female sex (*z* = 0.9, *P* = 0.3), baseline mean hypertensive patients (*z* = −0.3, *P* = 0.8), baseline left ventricular ejection fraction (LVEF) (*z* = −0.1, *P* = 0.9), and baseline mean diabetic patients (*z* = 1.7, *P* = 0.09) had no moderating effect on all-cause mortality for presence/absence of RAS inhibitors (see **Table 2, Supplemental Digital Content 1**, http://links.lww.com/AJT/A210). Visual inspection of the funnel plot showed no visible asymmetry supported by Eggers' linear regression (*t* = −1.89, *P* = 0.09) (see **Figure 6, Supplemental Digital Content 1**, http://links.lww.com/AJT/A210).

**Table 1.** Characteristics of included studies.

Study, year	Study design	Sample size		Age*		Female (%)	LVEF*	DM (%)	HTN (%)	HF (%)	MI (%)	Follow-up (year)
		RASI	Control	RASI	Control							
Amat-Santos et al^5^	RCT	94	92	82.5	83.3	58.1	60.4	21.25	58.05	38.7	7.5	1
Basile et al^11^	C^†^	105	105	83.51	83.48	29.55	54.1	30.45	—	35.7	—	2
Chen et al^12^	C	1736	2243	81.7	82.9	40.4	54.7	36.55	93	83.4	19.05	2
Cubeddu et al^13^	C	3172	5840	79.3	80.4	46.85	—	46.2	95.15	49.25	17.1	3
Fischer-Rasokat et al^14^	C^†^	626	626	82.1	81.9	54.35	61.35	32.65	84.25	79.1	10.95	3
Inohara et al^15^	C^†^	7948	7948	82.4	82.4	48.05	51.95	38.7	93.35	79.7	24.1	1
Kaewkes et al^16^	C	349	415	81.4	82.9	41.5	59	29	90	94	9.5	2
Klinkhammer et al^17^	C	71	98	77.8	80.1	41.5	57.1	41.5	86.5	46	0	1
Ledwoch et al^18^	C	225	98	80.7	79.8	45	52.05	22.4	87.6	48.1	13.95	3
Ochiai et al^19^	C	371	189	84.2	84.8	72.9	63.1	26.05	72.6	50.55	7.35	2
Phuong et al^20^	C	308	427	77.9	79.3	45.5	55.9	40.5	89.6	—	23.95	—
Rodriguez-Gabella et al^21^	C^†^	695	695	80.8	80.6	53.75	58.3	34.4	78.2	67.35	15.65	1
Tomii et al^22^	C	1634	613	81.8	81.9	50	55	26	84.7	68.3	13.75	1

* Mean.

† Matched.

C, cohort; DM, diabetes mellitus; HF, heart failure; HTN, hypertension; MI, myocardial infarction; RASI, renin-angiotensin aystem inhibitor; RCT, randomized controlled trial.

**FIGURE 1.**
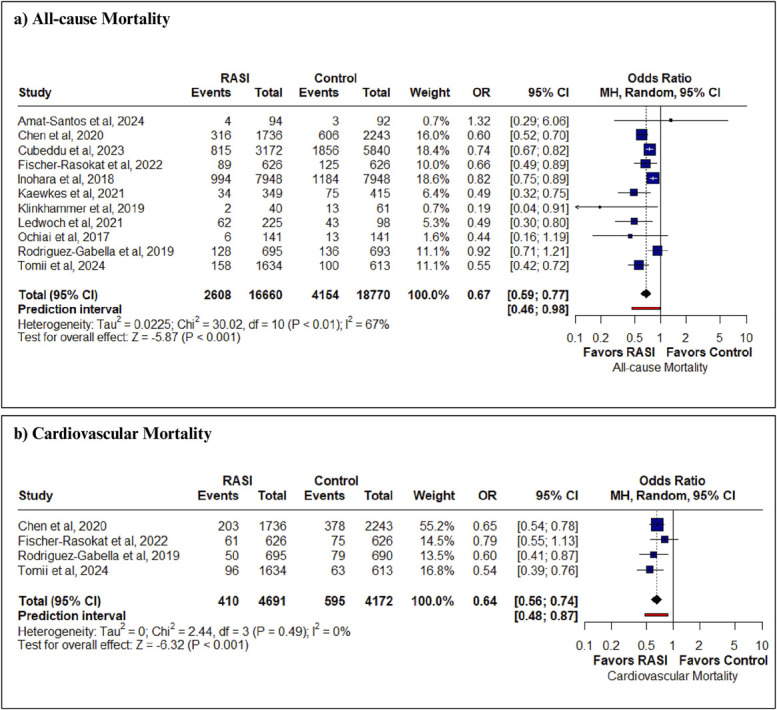
Forest plots of (A) all-cause mortality and (B) cardiovascular mortality.

Cardiovascular mortality was significantly lower in RAS inhibitors (OR 0.64; 95% CI, 0.56–0.74; *P* < 0.001; I^2^ = 0) compared to Control (Figure 1B). Risk of heart failure (OR 0.80; 95% CI, 0.62–1.04; *P* = 0.091), major bleeding (OR 0.88; 95% CI, 0.73–1.07; *P* = 0.21), aortic regurgitation (OR 1.09; 95% CI, 0.93–1.28; *P* = 0.27), mitral regurgitation (OR 0.99; 95% CI, 0.88–1.13; *P* = 0.92), acute kidney injury (OR 0.71; 95% CI, 0.47–1.07; *P* = 0.104), and stroke/TIA (OR 0.79; 95% CI, 0.49–1.28; *P* = 0.34) were comparable between the 2 groups (see **Figure 7, Supplemental Digital Content 1**, http://links.lww.com/AJT/A210).

In this review, the findings provide important evidence for the beneficial effects of RAS inhibitors in improving long-term clinical outcomes after TAVR. An observed reduction in cardiovascular mortality was noted, which can be attributed to the multifaceted cardioprotective mechanism of RAS inhibitors. By blocking the RAS, they can reduce the stress on the heart by lowering the left ventricular afterload, preventing fibrosis, and supporting myocardial remodeling.^23^ In addition, RAS inhibitors have anti-inflammatory effects that reduce aortic valve inflammation and calcification through pharmacologic inhibition of the angiotensin II pathway. This mechanism counteracts the systemic inflammation caused by TAVR, ultimately leading to improved survival rates and reduced risk of cardiovascular mortality.^24^ These results align with the results of the Bhat et al meta-analysis, supporting the beneficial role of RAS inhibitors after TAVR.^25^ A notable dose-dependent relationship between RAS inhibitor use and reduced mortality was reported in 2 studies,^14,18^ highlighting their critical role in post-TAVR management. These findings underscore the need for future studies on optimizing dosage strategies.

Despite a significant increase in survival benefit, no major improvements were found in other clinical outcomes, including heart failure, acute kidney injury, major bleeding, stroke/TIA, and aortic or mitral regurgitation. This discrepancy suggests that the benefits of RAS inhibitors in these clinical outcomes may be influenced by factors such as comorbidities, disease severity, and patient-specific factors. For example, the absence of its effect on bleeding or stroke signifies the selective mechanism of action of RAS inhibitors, which may not extend to these outcomes.

Despite the promising findings, some study limitations need to be considered. One major limitation is potential bias because of the predominance of observational studies with only 1 RCT part of the review. Further RCTs are warranted to assess and understand the precise impact of RAS inhibitors after TAVR.

In conclusion, this review provides strong evidence supporting the use of RAS inhibitors as a safe and effective strategy to improve clinical outcomes in patients undergoing TAVR. The strength of the study was due to the rigorous methodology and large, pooled sample size. Future studies should focus on dose optimization, timing, patient selection criteria, and combining RAS inhibitors with other treatments to maximize the benefits with limited risks and optimize outcomes in this patient population.

## Supplementary Material
