# Supplementary material for: Role of Renin–Angiotensin System Inhibitors After Transcatheter Aortic Valve Replacement: A Systematic Review and Meta-analysis
Source: Am J Ther. 2025 Jan 29;32(4):e382–5. doi: 10.1097/MJT.0000000000001897 (PMC12203980; doi:10.1097/MJT.0000000000001897)
Supplement: Supplementary file 1 [file ajt-32-e382-s001.docx]

**SUPPLEMENTAL MATERIALS**

**Role of Renin-Angiotensin System (RAS) Inhibitor following Transcatheter Aortic Valve Replacement (TAVR): a Systematic Review and Meta-Analysis**

**CONTENTS**

|  | **Page** |
| --- | --- |
| Supplemental Table 1: Search Strategy and Terms | 2, 3 |
| Supplemental Figure 1: PRISMA Flowchart | 4 |
| Supplemental Figure 2: Risk of Bias Assessment using RoB 2.0 | 5 |
| Supplemental Figure 3: Risk of Bias Assessment using ROBINS-I | 6 |
| Supplemental Figure 4: Leave-one-out Sensitivity Meta-analysis | 7 |
| Supplemental Figure 5: Sub-group Meta-analysis | 8 |
| Supplemental Table 02: Univariate Meta-Regression | 9 |
| Supplemental Figure 6: Funnel Plots and Trim-and-fill Analysis for Publication Bias | 10 |
| Supplemental Figure 7: Forest Plots of Secondary Outcomes | 11 |

**Supplemental Table 1: Search String**

| **Database** | **Search** | **Results** |
| --- | --- | --- |
| **PUBMED** | (((((((((((((((((((((((((((((((Angiotensin-Converting Enzyme Inhibitors[Mesh]) OR (Angiotensin Converting Enzyme Inhibitors)) OR (Enzyme Inhibitors, Angiotensin-Converting)) OR (Inhibitors, Angiotensin-Converting Enzyme)) OR (Inhibitors, Angiotensin Converting Enzyme)) OR (Inhibitors, Kininase II)) OR (Kininase II Antagonists)) OR (Kininase II Inhibitors)) OR (Angiotensin-Converting Enzyme Antagonists)) OR (Angiotensin Converting Enzyme Antagonists)) OR (Enzyme Antagonists, Angiotensin-Converting)) OR (Antagonists, Angiotensin-Converting Enzyme)) OR (Antagonists, Angiotensin Converting Enzyme)) OR (Antagonists, Kininase II)) OR (Inhibitors, ACE)) OR (ACE Inhibitors)) OR (Angiotensin I-Converting Enzyme Inhibitors)) OR (Angiotensin I Converting Enzyme Inhibitors)) OR (Angiotensin Converting Enzyme Inhibitor)) OR (ACE Inhibitor)) OR (Inhibitor, ACE)) OR (Angiotensin I-Converting Enzyme Inhibitor)) OR (Angiotensin I Converting Enzyme Inhibitor)) OR (Angiotensin-Converting Enzyme Inhibitor)) OR (Enzyme Inhibitor, Angiotensin-Converting)) OR (Inhibitor, Angiotensin-Converting Enzyme)) OR (Kininase II Inhibitor)) OR (II Inhibitor, Kininase)) OR (Inhibitor, Kininase II)) OR (((((((((((((((Angiotensin Receptor Antagonists[Mesh]) OR (Antagonists, Angiotensin Receptor)) OR (Receptor Antagonists, Angiotensin)) OR (Angiotensin Receptor Antagonist)) OR (Antagonist, Angiotensin Receptor)) OR (Receptor Antagonist, Angiotensin)) OR (Angiotensin Receptor Blocker)) OR (Blocker, Angiotensin Receptor)) OR (Receptor Blocker, Angiotensin)) OR (Angiotensin Receptor Blockers)) OR (Receptor Blockers, Angiotensin)) OR (Angiotensin II Receptor Antagonists)) OR (Angiotensin II Receptor Antagonist)) OR (Angiotensin II Receptor Blocker)) OR (Angiotensin II Receptor Blockers))) OR (((((((Renin Inhibitors[Mesh]) OR (Inhibitors, Renin)) OR (Renin Inhibitor)) OR (Direct Renin Inhibitor)) OR (Direct Renin Inhibitors)) OR (Inhibitors, Direct Renin)) OR (Renin Inhibitors, Direct))) AND ((Transcatheter Aortic Valve Replacement[Mesh]) OR (Transcatheter Aortic Valve Implantation)) | 55 |
| **EMBASE** | ('transcatheter aortic valve implantation'/exp OR 'duravr tavr' OR 'tavi' OR 'percutaneous aortic valve implantation' OR 'percutaneous aortic valve replacement' OR 'trans-apical aortic valve implantation' OR 'trans-apical aortic valve replacement' OR 'trans-arterial aortic valve implantation' OR 'trans-arterial aortic valve replacement' OR 'trans-catheter aortic valve implantation' OR 'trans-catheter aortic valve replacement' OR 'trans-cutaneous aortic valve implantation' OR 'trans-cutaneous aortic valve replacement' OR 'trans-femoral aortic valve implantation' OR 'trans-femoral aortic valve replacement' OR 'transapical aortic valve implantation' OR 'transapical aortic valve replacement' OR 'transarterial aortic valve implantation' OR 'transarterial aortic valve replacement' OR 'transcatheter aortic valve implantation' OR 'transcatheter aortic valve replacement' OR 'transcutaneous aortic valve implantation' OR 'transcutaneous aortic valve replacement' OR 'transfemoral aortic valve implantation' OR 'transfemoral aortic valve replacement') AND ('dipeptidyl carboxypeptidase inhibitor'/exp OR 'ace inhibitor' OR 'angiotensin converting enzyme inhibiting agent' OR 'angiotensin converting enzyme inhibitor' OR 'angiotensin converting enzyme inhibitors' OR 'angiotensin i converting enzyme inhibitor' OR 'angiotensin-converting enzyme inhibitors' OR 'converting enzyme inhibitor' OR 'dipeptidyl carboxypeptidase i inhibitor' OR 'dipeptidyl carboxypeptidase inhibitor' OR 'kininase ii inhibitor' OR 'peptidyl dipeptidase inhibitor' OR 'peptidyldipeptide hydrolase inhibitor' OR 'angiotensin receptor antagonist'/exp OR 'angiotensin ii receptor antagonist' OR 'angiotensin ii receptor antagonists' OR 'angiotensin ii receptor blocker' OR 'angiotensin ii receptor blockers' OR 'angiotensin ii receptor blocking agent' OR 'angiotensin ii receptor blocking agents' OR 'angiotensin receptor antagonist' OR 'angiotensin receptor antagonists' OR 'angiotensin receptor blocker' OR 'angiotensin receptor blockers' OR 'angiotensin receptor blocking agent' OR 'angiotensin receptor blocking agents' OR 'renin inhibitor'/exp OR 'inhibition, renin' OR 'inhibitor, renin' OR 'renin inhibition' OR 'renin inhibitor' OR 'renin inhibitors') | 597 |
| **Cochrane CENTRAL** | **#1** MeSH descriptor: [Angiotensin-Converting Enzyme Inhibitors] explode all trees  **#2** MeSH descriptor: [Angiotensin II Type 2 Receptor Blockers] explode all trees  **#3** angiotensin receptor blocker  **#4** renin-angiotensin inhibitor  **#5** MeSH descriptor: [Renin Inhibitors] explode all trees  **#6** #1 OR #2 OR #3 OR #4 OR #5  **#7** MeSH descriptor: [Transcatheter Aortic Valve Replacement] explode all trees  **#8** transcatheter aortic valve implantation  **#9** #7 OR #8  **#10** #6 AND #9 | 7 |

**Supplemental Figure 01: PRISMA Flowchart:**

**
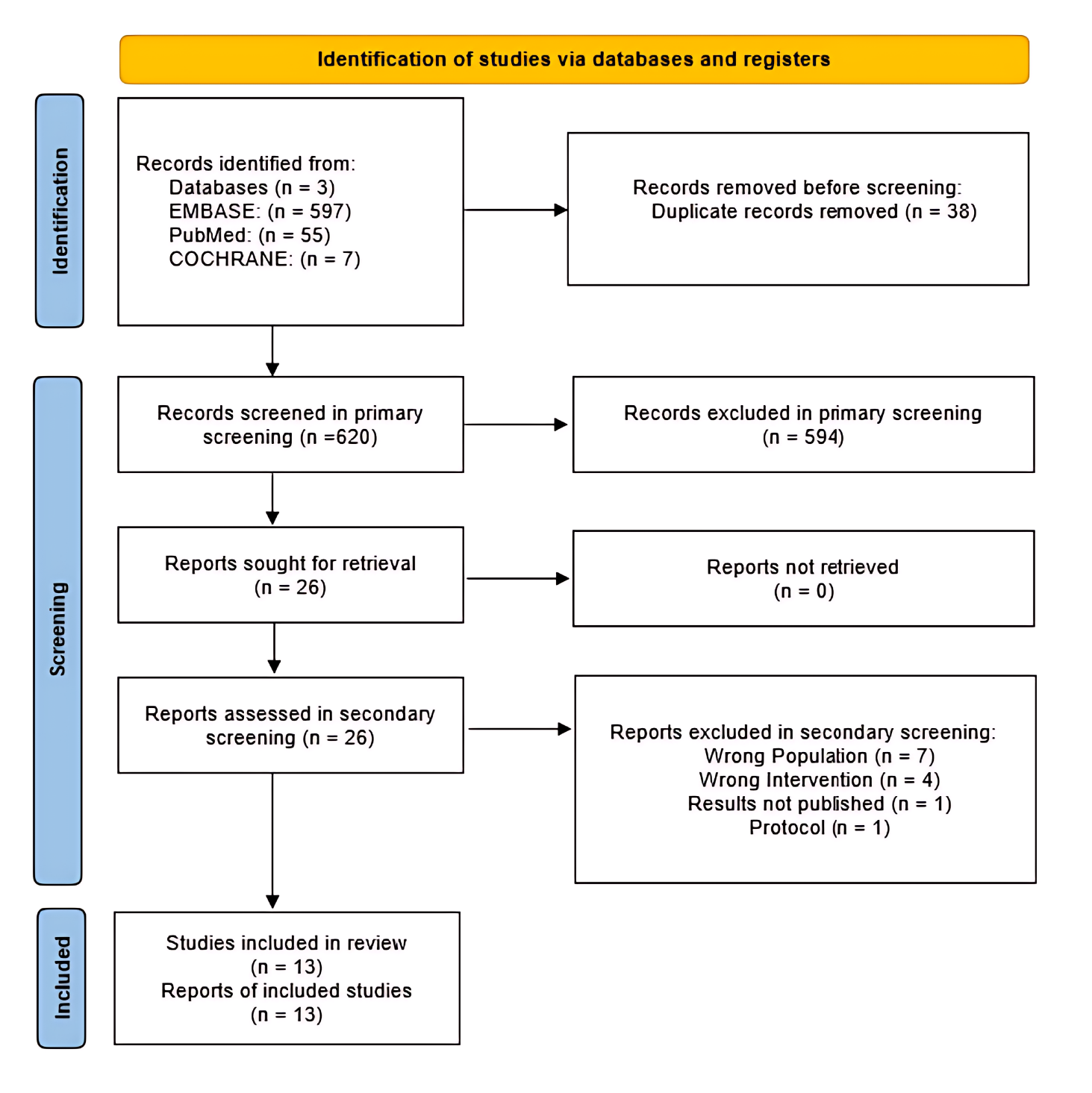
**

**Supplemental Figure 02: Risk of Bias Assessment of randomized controlled trials using RoB 2.0**

**
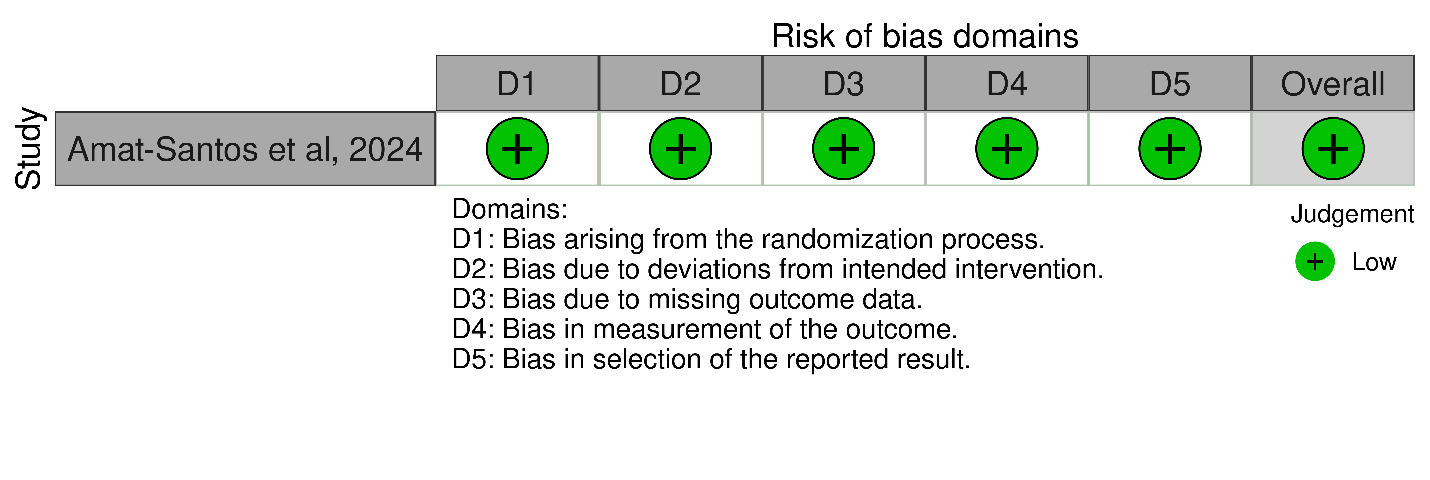
Traffic plot:**

**
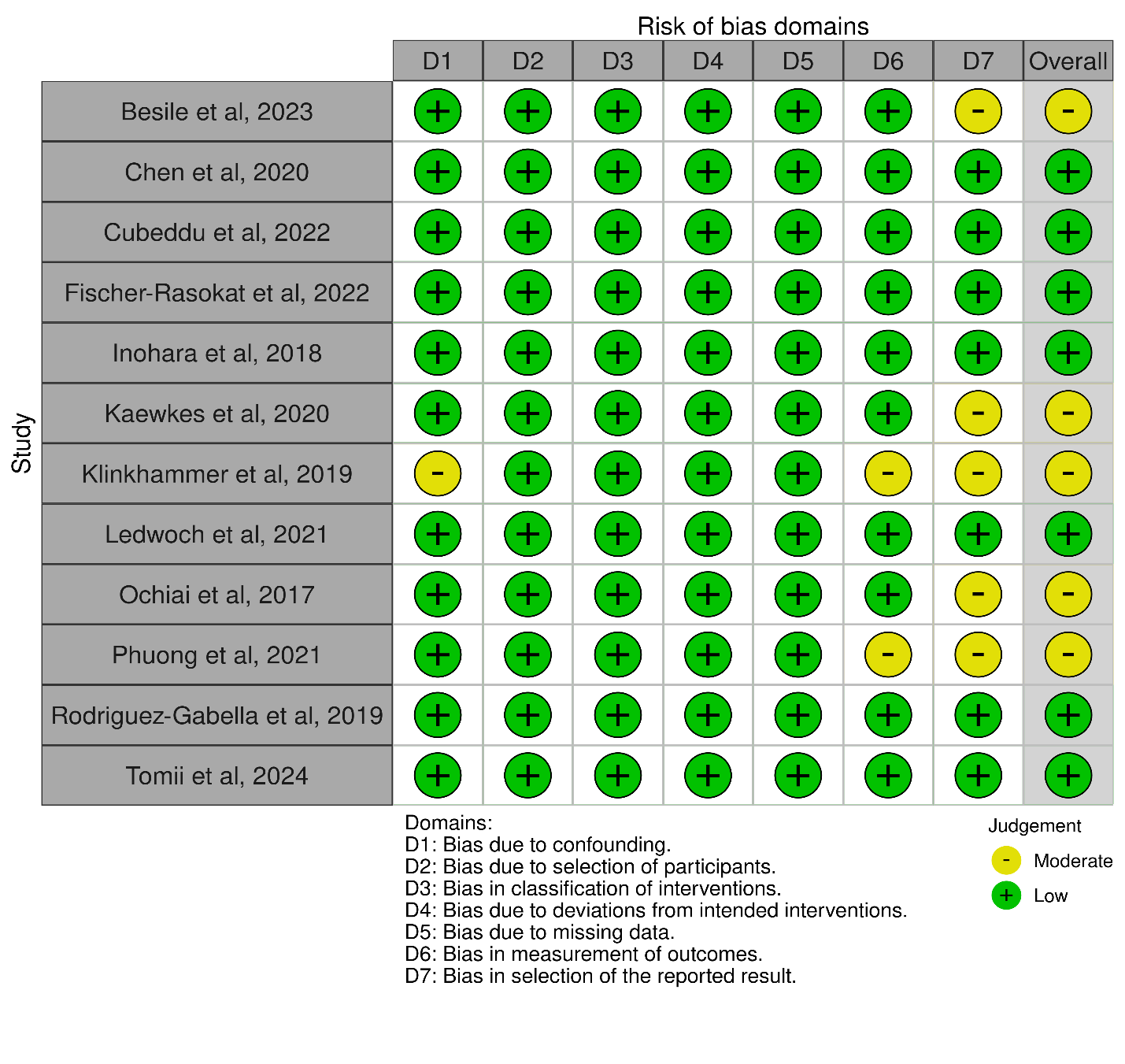
Supplemental Figure 03: Risk of Bias Assessment of non-randomized studies of interventions
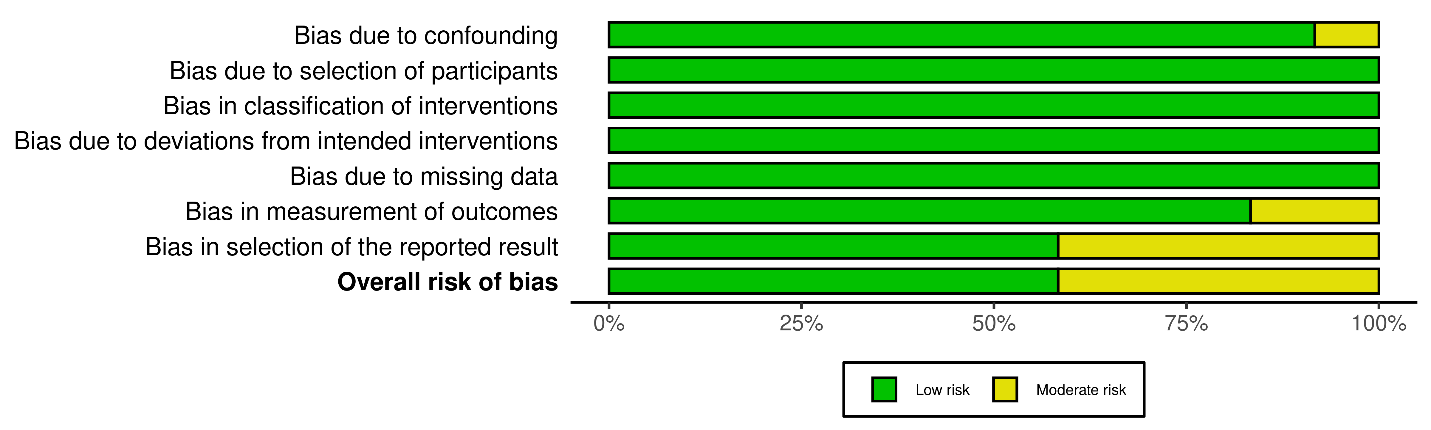
 using ROBINS-I
Traffic plot and risk of bias summary:**

**Supplemental Figure 04: Leave-one out Sensitivity Meta-analysis of primary outcome:**

**
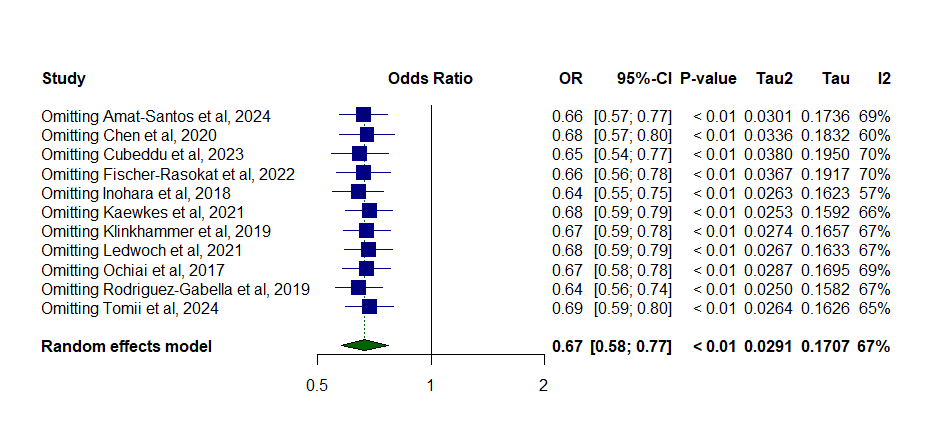
**

**Supplemental Figure 05: Sub-group Meta-analysis of primary outcome based on follow-up duration:**

**
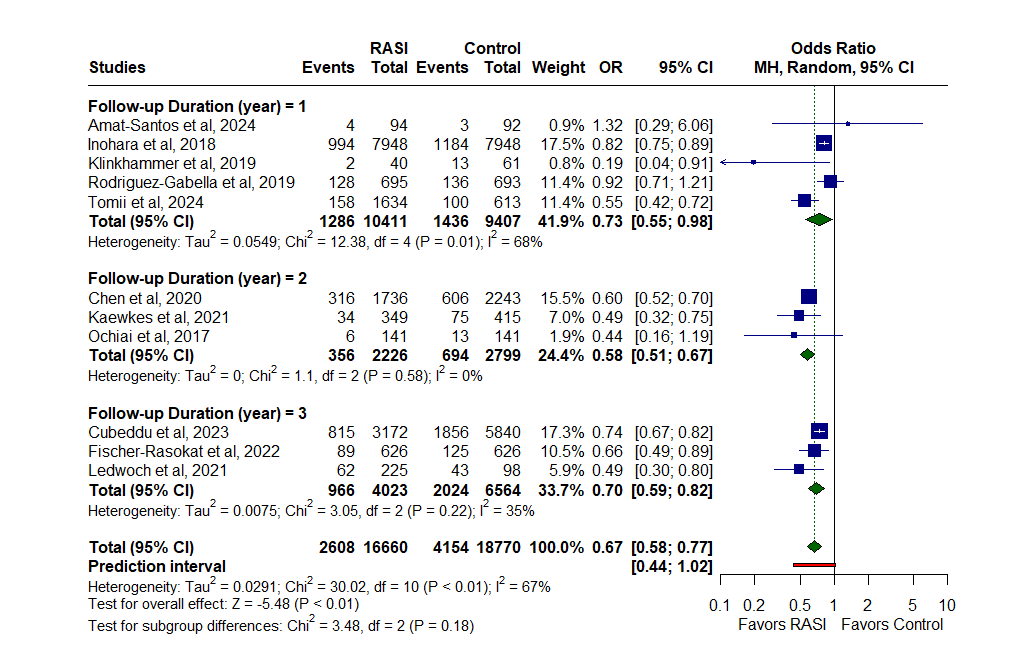
**

**Supplemental Table 02: Univariate Meta-Regression of Primary Outcome**

| **Moderator** | **z value** | **95% CI** | **R^2^** | ***p* value** |
| --- | --- | --- | --- | --- |
| Publication year | -0.9 | -0.1, 0.03 | 16.38% | 0.34 |
| Age | -0.3 | -0.16, 0.11 | 0% | 0.74 |
| Female sex | 0.94 | -0.01, 0.03 | 8.63% | 0.34 |
| Baseline mean hypertensive patients | -0.28 | -0.02, 0.02 | 0% | 0.78 |
| Baseline LVEF | -0.14 | -0.05, 0.05 | 0% | 0.89 |
| Baseline mean diabetic patients | 1.68 | -0.003, 0.03 | 31.73% | 0.09 |

**Supplemental Figure 06: Funnel plot and Egger’s Test**

**
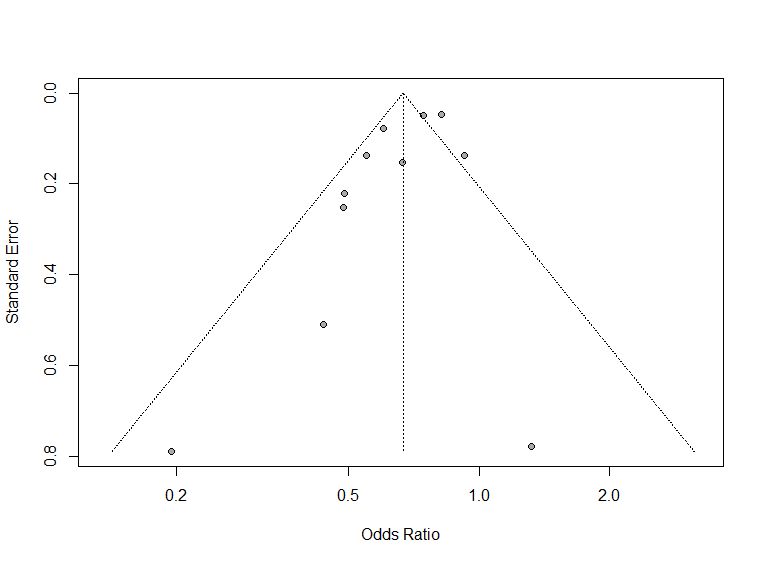
**

| **Intercept** | **95% CI** | **t** | **P value** |
| --- | --- | --- | --- |
| -1.36 | -2.77 to 0.05 | -1.89 | 0.092 |

**
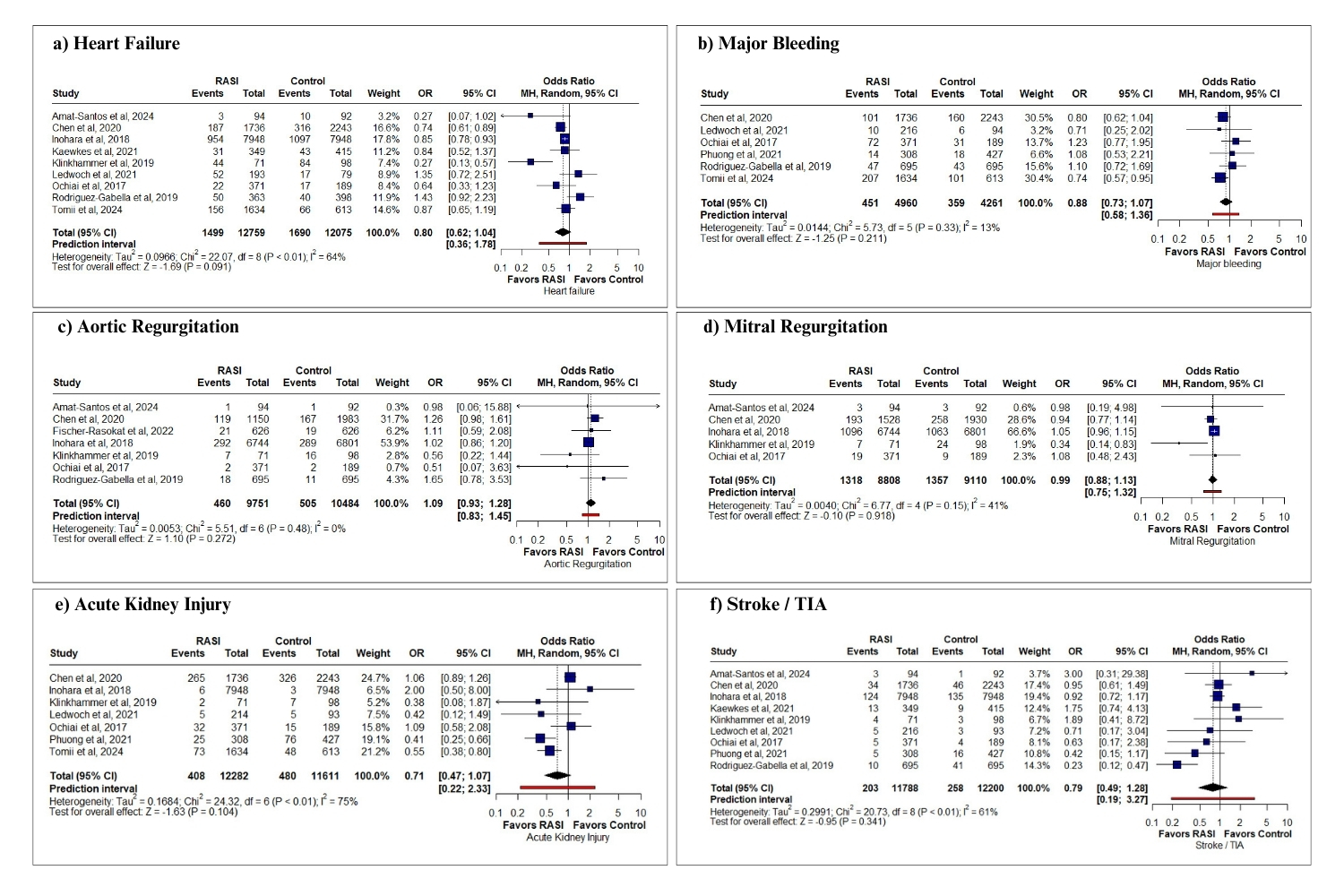
 Supplemental Figure 07: Forest Plots of Secondary Outcomes:**
